# Rotation of both X- and Y-axes is a predictive confounder of ulnar nerve injury and open reduction in pediatric lateral flexion supracondylar humeral fractures: A retrospective cohort study

**DOI:** 10.3389/fped.2022.962521

**Published:** 2022-10-04

**Authors:** Jun Sun, Jing Shan, Lian Meng, Tianjing Liu, Enbo Wang, Guoqiang Jia

**Affiliations:** ^1^Children's Hospital of Anhui Province, Hefei, China; ^2^Pediatric Orthopedics, Shengjing Hospital of China Medical University, Shenyang, China

**Keywords:** supracondylar fracture of the humerus, SCHFs, lateral flexion, ulnar nerve injury, open reduction, fracture lever

## Abstract

**Background:**

Rotation of the distal fragment often occurs in flexion-type supracondylar humerus fractures (SCHFs), potentially leading to ulnar nerve injury (UNI) and open reduction. We analyzed the correlation between the rotations and UNI or open reduction and then assessed the risk factors associated with these rotations.

**Methods:**

Data of Wilkins type III lateral flexion SCHFs were collected over a 10-year time period (1 January 2012 to 31 December 2021) in Children's Hospital of Fudan University Anhui Hospital. We defined the rotation of the distal fragment on the coordinate axis as two types, IIIA (*X*-axis rotation) and IIIB (the rotation of both *X*- and *Y*-axes) on X-ray radiography. Demographic data, the incidence of the two-type rotation, odds ratios (ORs) of UNI and open reduction, and risk factors of the rotation of both *X*- and *Y*-axes were analyzed.

**Results:**

Totally, 152 patients were found (50 with IIIA vs. 102 IIIB). The UNI rate was 13%, and the open reduction rate was 22%. The UNI rate of the IIIB was five-fold higher than that of the IIIA [OR, 5.143; 95% confidence interval (CI), 1.414–23.125; *p* = 0.019], and the open reduction rate of the IIIB was nearly five-fold higher than that of the IIIA (OR, 4.729, 95%CI, 1.584–14.495; *p* = 0.003). In these two types, patients with UNI had a higher risk of open reduction than those without UNI (OR, 9.816; 95%CI, 3.503–27.508; *p* = 0.001). In the multiple regression analysis, a high level of fracture was identified as a risk factor for the rotation of both *X*- and *Y*-axes.

**Conclusion:**

Type IIIB lateral flexion-type SCHFs have higher rates of UNI and open reduction, and a high level of fracture is a risk factor associated with this type.

## Introduction

Supracondylar fracture of the humerus (SCHFs) is one of the most common elbow fractures in children, and flexion-type SCHFs accounts for 2%−4% of all SCHFs (1–3). Flexion-type SCHFs are usually laterally deviated, which are divided into three subtypes by Wilkins in 1990, according to the mirror image relationship with the classical extension-type proposed by Gartland (4, 5). Type I is minimally displaced with both anterior and posterior cortex integrity; type II is a simple anterior displacement with anterior cortex integrity; and type III is displaced without cortex integrity (5). The classical treatment algorithm, which was recommended in the flexion type, was similar to the extension type from cast immobilization to open reduction and pinning (6, 7).

Flexion-type SCHFs are often associated with higher risks of ulnar nerve injury (UNI) than extension-type (8–11). For a higher incidence of UNI, the two major reasons are: (1) the direction of anterior lateral translocation of the distal fragment leading to excessive tension of ulnar nerves in the posterior medial side and (2) the fractured fragment posssibly being shaped as a medial spike, which can compress or puncture ulnar nerves. For a higher incidence of open reduction, the fragment spike can puncture muscles and the ulnar nerve can be an entrapment, which does not facilitate closed reduction. In addition, several surgical techniques have been used, but open reduction is still unavoidable in some severe rotational cases (12–14). According to the anatomical rotation characteristics of type III flexion SCHFs, the distal fragment would rotate on the X-axis or both *X*- and *Y*-axes. However, the correlation between the two rotations and UNI or open reduction remains unclear.

Therefore, we hypothesized the two rotations of the distal fragment as two new subtypes of type III flexion SCHF and analyzed the correlation between the rotations and UNI or open reduction and then assessed the risk factors associated with the rotations of both *X*- and *Y*-axes.

## Materials and methods

### Subjects

This study was approved by the institutional review board of Children's Hospital of Fudan University, Anhui, in accordance with the Declaration of Helsinki, and the consent of patients or their guardians was obtained.

In total, 4,831 patients with SCHFs in our hospital between January 2012 and December 2021 were screened. According to inclusion and exclusion criteria, 152 patients were included. Gender, domination hand, body mass index (BMI), age, and the level of fracture were analyzed. The inclusion criteria were flexion-type III SCHFs; available preoperative initial anteroposterior (AP) and lateral X-ray radiographs; and medical records including height, weight, medical history, and operation documents. The exclusion criteria were SCHFs with an ulnar deviation; manipulation before original X-ray radiography; patients' or their guardians' refusal; and open fractures.

### Classification and analysis of x-ray radiography

#### The definition of the coordinate axis

**Figure 1:**
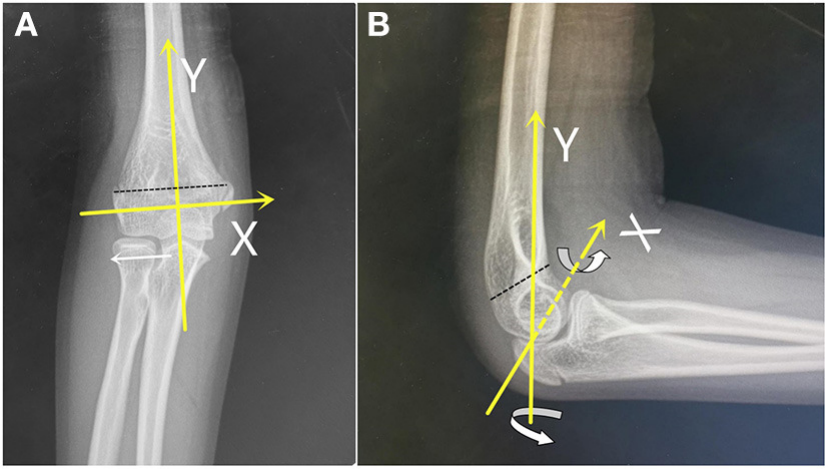
Translocation and rotation schema on the coordinate axis (yellow line) on normal anteroposterior (AP) and lateral radiographs. The *Y*-axis is parallel to the humeral shaft, the *X*-axis is perpendicular to the *Y*-axis crossing both supracondylar, and the black dotted line indicates the fracture line. In **(A)**, the white arrow indicates lateral translocation; in **(B)**, the rotate arrows indicate the rotation along the *X*- or *Y*-axes of the distal fragment.

In this study, the coordinate axis was drawn on AP and lateral films of SCHFs, and the center of the normal olecranon fossa location was defined as the coordinate center. The longitudinal axis of the humerus was defined as the *Y*-axis, and the line perpendicular to the *Y*-axis through the medial and lateral epicondylar was defined as the *X*-axis. The distal fracture fragment rotations were determined along the *X*- and *Y*-axes on the coordinate axis with reference to the proximal humeral shaft (Figure 1). Patients were divided into two types by the spatial rotation characteristics of the distal fracture fragment on preoperative initial AP and lateral X-ray images.

The fracture levels were classified as high or low according to Kang's description of the fracture line above or below the isthmus of the distal humerus (15), respectively.

#### The classification of two subtypes

The final results of the two types were identified by two senior orthopedic specialists.

**Figure 2:**
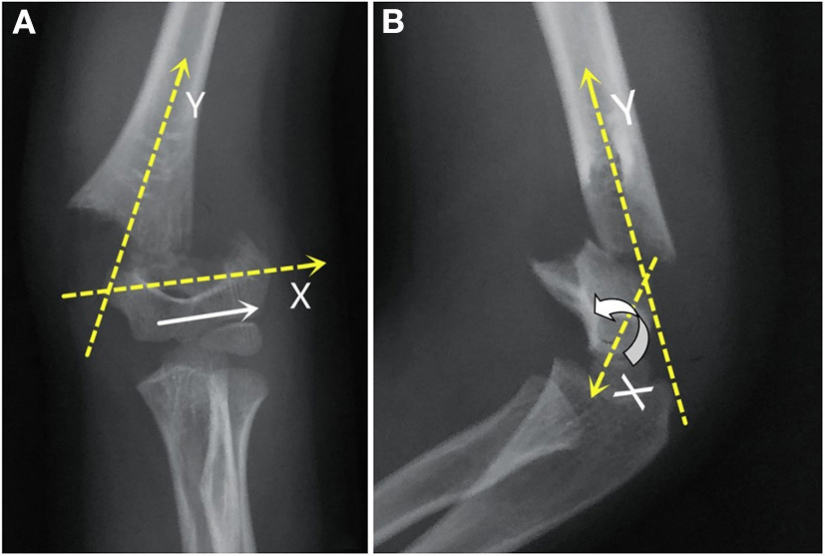
In the AP view **(A)**, the distal fragment (white arrow) was anterior-lateral translocation. In the lateral view **(B)**, the distal fragment was the rotation on the *X*-axis without an obvious *Y*-axis rotation.

**Figure 3:**
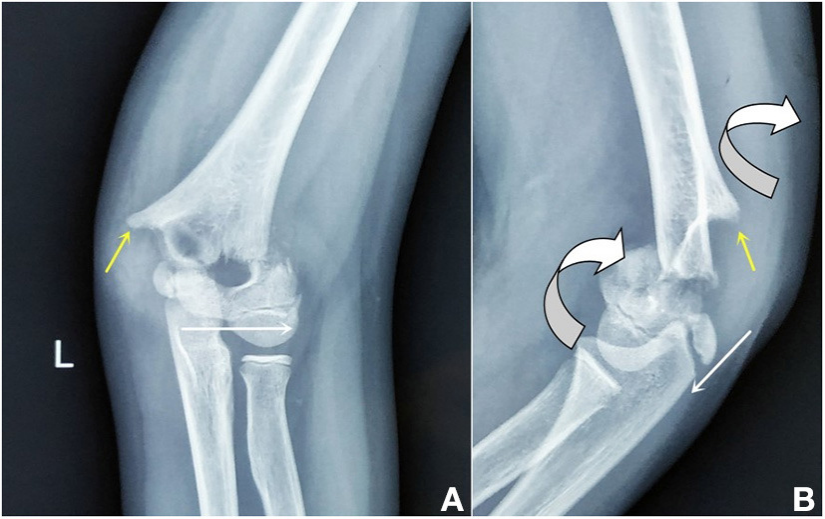
In the AP view **(A)**, the distal fragment (white arrow) was anterior-lateral translocation. In the lateral view **(B)**, the distal fragment was obviously rotation both on *X*- and *Y*-axes (rotation arrow), and the proximal fragment spike was obvious (yellow arrow).

- IIIA: The distal fracture fragment was with only a rotation on the *X*-axis, and there was no proximal metaphyseal medial spike on a lateral film (AP tilt, Figure 2).
- IIIB: The distal fracture fragment was with a rotation on both *X*- and *Y*-axes, and there was usually a proximal metaphyseal medial spike on a lateral film (Figure 3).

### Statistical analysis

Statistical analysis was conducted using SPSS version 24.0 (IBM, Armonk, New York, USA). Statistical methods included standard descriptive summaries of demographic data; these were analyzed using the Chi-squared test, thew Fisher's exact test, or the two-sample *t*-test. Regression analysis was used to identify the risk factors of type IIIB.

## Results

### Demographic data

The average age was 8.78 ± 2.51 (2–15) years in 152 children with lateral flexion SCHFs. Of these, 50 were type IIIA and 102 were type IIIB. There was no significant difference between the two types in gender, domination hand, and BMI. The age and fracture level had a significant difference between the two groups (*p* = 0.015; *p* = 0.001; see Table 1).

**Table 1:** Injury characteristics of 152 skeletally immature patients with lateral flexion supracondylar humerus fractures (SCHF).

**Characteristics**	**IIIA (50 cases)**	**IIIB (102 cases)**	***t* or *χ^2^***	***p*-Value**
Male gender, *n* (%)	29 (58)	56 (55)	0.031	0.718^‡^
Domination hand, *n* (%)	33 (66)	58 (57)	1.166	0.280^‡^
Mean BMI (kg/m^2^)	20.87 ± 5.19	21.83 ± 5.12	1.093	0.276^†^
Mean age at surgery (years)	8.08 ± 2.30	9.13 ± 2.54	2.462	0.015^†^
Fracture level (high:low)	29:21	85:17	11.484	< 0.001^‡^
Ulna nerve injury, *n* (%)	2 (4)	18 (18)	5.469	0.019^‡^
Open reduction, *n* (%)	4 (8)	30 (29)	8.858	0.001^‡^

^†^Student *t*-test.

^‡^χ^2^ test.

### UNI rate

Among the 21 patients with nerve injury, 20 (95%) were UNI and one had a radial nerve injury. Among the 20 cases, two (10%) were classified as type IIIA and 18 (90%) were type IIIB. The odds ratio (OR) of UNI showed that the type IIIB group was five-fold higher than the IIIA group [OR, 5.143; 95% confidence interval (CI), 1.414–23.125; *p* = 0.019]. The results are shown in Table 2.

**Table 2:** Univariate odds of SCHF in different rotation fracture types.

**Type**	**Ulnar nerve injury**			**Open reduction**	
	**Referent**	**OR (CI 95%)**	***p*-Value**	**OR (CI 95%)**	***p*-Value**
IIIB	IIIA	5.143 (1.414–23.125)	0.019	4.792 (1.584–14.495)	0.003

### Open reduction rate

Open reduction was performed in 34 patients (22%), including four cases of type IIIA and 30 cases of type IIIB (30/34). The OR of open reduction showed that the type IIIB group was nearly five-fold higher than the type IIIA group (OR, 4.729; 95%CI, 1.584–14.495; *p* = 0.003; Table 2). There were 13 cases (IIIA, 1; IIIB, 12) with UNI in the 34 patients receiving open reduction, and seven cases with UNI in the other 118 patients treated by closed reduction. In all patients, patients with UNI had a significantly higher risk of open reduction than those without UNI (OR, 9.816; 95%CI, 3.503–27.508; *p* = 0.001). Type IIIB patients with UNI had a higher risk of open reduction than type IIIA patients with UNI (OR, 6.000; 95%CI, 0.509–70.668; *p* = 0.264).

### Risk factors associated with IIIB rotation

Totally, there were 114 cases of high-level and 38 cases of low-level SCHFs. A high level of incidence was 58% in IIIA and 83% in IIIB. Age and the level of fracture were significantly different in the two groups (IIIA and IIIB). According to the results of multiple logistic regression analysis, a high level of fracture was identified as an independent risk factor of type IIIB rotation (OR, 3.210; 95% CI, 1.470–7.011; *p* = 0.003; Table 3).

**Table 3:** The results of multiple logistic regression analysis of risk factors associated with type IIIB rotation.

**Variables**	**IIIB rotation**		
	**OR**	**CI 95%**	***p*-Value**
Fracture level	3.210	1.470–7.011	0.003
Age	1.155	0.994–1.343	0.060

## Discussion

Type III flexion SCHF is an important fracture in pediatric orthopedic clinical practice, and most of them are laterally deviated (16). It has attracted special attention from surgeons due to the high rates of UNI and open reduction, especially for those with a medial spike in the proximal fragment (8–11, 16, 17). We classified the rotation of the *X*-axis or both *X*- and *Y*-axes of the distal fracture fragment into two subtypes of type III flexion SCHF, and we proposed that these two subtypes could be helpful for an orthopedic to evaluate UNI and make a better decision between close and open reduction in daily clinical practice.

Ulnar nerve injury is caused by several reasons: the direction of anterior lateral translocation of the distal fragment leading to excessive tension of the ulnar nerve on the posterior medial side; and the proximal medial metaphyseal spike, which posteriorly compresses or punctures the ulnar nerve and in some cases can be even entrapped between the two fragments in flexion SCHFs (11, 16, 17). An early and precise evaluation is required for UNI; traditionally, the clipping test and the Forment's sign are clinically used. However, these two traditional methods could be affected by tissue swelling, pain, and muscle impact. Therefore, our subtype could be helpful in these situations. In our study, we also found that the total UNI rate in type III flexion SCHFs was 13%, and the UNI rate in the type IIIB group (18%) was increased significantly than that in type IIIA (4%). Due to the lack of patients with type III flexion SCHF, few previous studies focused on the UNI rate in this type. Usually, UNI rates in the total flexion SCHF have been reported with a wide range of 10.5%−26% (8, 9, 11, 18, 19). In a meta-analysis study, the UNI rate was calculated as 14% in flexion SCHFs, and type II and type III patients were not separated for an analysis (20). To our knowledge, our study presented an innovative and detailed understanding of type III flexion SCHFs. According to the results of our subtype UNI rate, we suggested that more attention should be paid to patients with subtype IIIB fracture for UNI. Furthermore, due to the instability of fractured fragments in both *X*- and *Y*-axes, closed reduction overtime should be avoided in this subtype because it could cause iatrogenic UNI (21).

Open reduction is an alternative treatment in SCHFs in case of failure of closed reduction. According to the study by Flynn et al. (9), the flexion type had a 15-fold higher risk of open reduction than the extension type; furthermore, we found in type III that type IIIB had a higher incidence of open reduction. Type IIIB flexion SCHF normally comes with a rotated proximal fragment spike, which detaches from the periosteum sheath and pierces the triceps muscle. Although some techniques are used in closed reduction, such as “joystick” and “push–pull,” the proximal spike may still remain irreducible or even have unsatisfactory alignment after reduction, which could cause restricted elbow function in older children with limited distal humerus remodeling and cubitus varus (22, 23). Based on our open reduction rates in the two subtypes, we thought that subtype IIIB had more unstable fractured fragments, which were more difficult to reduce by closed reduction. This is because, in type IIIB, most of the proximal fragment spikes rotated toward the triceps muscle and some compressed the ulnar nerve, and this led to swelling of the nerve epithelium, tortuous bleeding, and even breaking off of a few nerve fiber bundles in our study. Furthermore, we found four cases (4/34) with ulnar nerve entrapment in a fracture gap that impedes reduction, which was similar to Steinman's finding (11), and all of the four cases were in the type IIIB group. Therefore, our findings could provide more confidence for orthopedic surgeons in decision-making of reduction of type IIIB flexion SCHFs.

Additionally, some studies showed that UNI could increase the incidence of open reduction (8–11, 18). A 6.7-fold higher risk of open reduction was found in patients with UNI than in those without UNI (9). These are similar to our finding, which found an almost 10-fold higher risk of open reduction in the UNI group than in the non-UNI group, and most of the patients with UNI (12/13) were in the type IIIB group.

Furthermore, we found that a high level of fracture was the only risk factor for type IIIB, which may be related to anatomical characteristics of the distal humerus in the transverse fracture. A high level of fracture usually has a smaller fracture “contact area” than the low type, which decreases the force of sliding friction and is more unstable (24, 25). Therefore, type III flexion SCHFs with a high level of fracture are more likely to combine with the rotation of both *X*- and *Y*-axes.

This study has some limitations. First, this is a retrospective single-center study and lacks long-term follow-up of UNI outcomes and elbow function. Second, patients were difficult to homogenize on a nonstandard X-ray. Finally, the sample size of patients with UNI or open reduction is not large enough to analyze the risk factors for UNI or open reduction.

In conclusion, lateral flexion-type type IIIB SCHFs have higher rates of UNI and open reduction, and a high level of fracture is a risk factor associated with this type. Our findings could support further studies on a deeper understanding of type III flexion SCHF in UNI or open reduction and on analyzing the risk factors of them in both subtypes. Thus, prospective multi-center studies are needed in the future.

## Data availability statement

The original contributions presented in the study are included in the article/supplementary material, further inquiries can be directed to the corresponding author.

## Ethics statement

The studies involving human participants were reviewed and approved by Children's Hospital of Fudan University Anhui Hospital. Written informed consent to participate in this study was provided by the participants' legal guardian/next of kin. Written informed consent was obtained from the individual(s), and minor(s)' legal guardian/next of kin, for the publication of any potentially identifiable images or data included in this article.

## Author contributions

JSu wrote the article. JSh and LM collected data and analyzed statistics. TL and EW reviewed the article. GJ designed and reviewed the article. All authors contributed to the article and approved the submitted version.

## Conflict of interest

The authors declare that the research was conducted in the absence of any commercial or financial relationships that could be construed as a potential conflict of interest.

## Publisher's note

All claims expressed in this article are solely those of the authors and do not necessarily represent those of their affiliated organizations, or those of the publisher, the editors and the reviewers. Any product that may be evaluated in this article, or claim that may be made by its manufacturer, is not guaranteed or endorsed by the publisher.
